# 67553 Exoskeleton dynamics alter upper-limb coordination in a virtual reality reaching task

**DOI:** 10.1017/cts.2021.461

**Published:** 2021-03-30

**Authors:** Alexander Brunfeldt, Alexander Dromerick, Barbara Bregman, Peter Lum

**Affiliations:** 1 Georgetown University; 2 The Catholic University of America

## Abstract

ABSTRACT IMPACT: This proof-of-concept study demonstrates that systematically altering limb dynamics with two exoskeleton devices alters ingrained, bilateral upper-limb coordination with the potential to rehabilitate functional reaching in chronic stroke survivors OBJECTIVES/GOALS: Advances in virtual reality and exoskeleton technologies have allowed researchers to alter upper limb coordination with more precision than ever before. The goal of this study was to systematically enhance the use of the nondominant limb during a bimanual reaching task, with an eye towards improving rehabilitative strategies post stroke. METHODS/STUDY POPULATION: Healthy, right-handed volunteers performed a bimanual reaching task in virtual reality (VR) space while simultaneously moving under the influence of two exoskeleton devices. The VR task had participants move a shared cursor, displayed at the midpoint between the hands, to targets arranged at shoulder and eye levels and located at 70% of full arm extension. Two exoskeleton devices applied either resistive torque to the dominant limb or assistive torque to the non-dominant limb. Three-dimensional hand position data were recorded at 50 Hz and analyzed offline. The primary outcome measure was relative contribution, calculated as the ratio of dominant/non-dominant displacement. RESULTS/ANTICIPATED RESULTS: Preliminary results from 3 participants showed that during baseline trials, when no torque was applied by the exoskeletons, relative contribution was 50.6% in favor of the dominant hand, with the dominant hand reaching on average 1.1cm farther than the left. When the exoskeletons resisted movement in the dominant limb while simultaneously assisting movement in the non-dominant limb, relative contribution was 49.7% indicating an increase in non-dominant limb usage. Further analysis showed that this effect was driven by one participant who reached 3.7cm farther with her non-dominant hand compared to baseline. DISCUSSION/SIGNIFICANCE OF FINDINGS: These pilot data suggest our testing platform is capable of altering normal coordination patterns and is likely the result of participants adopting an optimal control strategy imposed by the shared cursor. These findings will form the basis for a rehabilitation intervention to promote the use of the paretic limb in chronic stroke survivors.

